# Herpesviruses in Reptiles

**DOI:** 10.3389/fvets.2021.642894

**Published:** 2021-05-05

**Authors:** God'spower Richard Okoh, Paul F. Horwood, David Whitmore, Ellen Ariel

**Affiliations:** Division of Tropical Health and Medicine, College of Public Health, Medical and Veterinary Sciences, James Cook University, Townsville, QLD, Australia

**Keywords:** herpesviruses, reptiles, fibropapillomatosis, taxonomy, pathogenesis, pathology, epidemiology

## Abstract

Since the 1970s, several species of herpesviruses have been identified and associated with significant diseases in reptiles. Earlier discoveries placed these viruses into different taxonomic groups on the basis of morphological and biological characteristics, while advancements in molecular methods have led to more recent descriptions of novel reptilian herpesviruses, as well as providing insight into the phylogenetic relationship of these viruses. Herpesvirus infections in reptiles are often characterised by non-pathognomonic signs including stomatitis, encephalitis, conjunctivitis, hepatitis and proliferative lesions. With the exception of fibropapillomatosis in marine turtles, the absence of specific clinical signs has fostered misdiagnosis and underreporting of the actual disease burden in reptilian populations and hampered potential investigations that could lead to the effective control of these diseases. In addition, complex life histories, sampling bias and poor monitoring systems have limited the assessment of the impact of herpesvirus infections in wild populations and captive collections. Here we review the current published knowledge of the taxonomy, pathogenesis, pathology and epidemiology of reptilian herpesviruses.

## Introduction

Reptiles are a group of vertebrates (class Reptilia) that are adapted to a broad range of terrestrial and aquatic environments (1, 2). The group comprises over 11,000 extant species placed in four orders, namely: Testudines (turtles, tortoises, and terrapins); Squamata (lizards, snakes, and worm lizards); Crocodilia (crocodiles, alligators, gharials, and caimans); and Rhynchocephalia (tuatara) (3). Reptiles constitute an integral part of the natural ecosystem and play the roles of both pollinators and predators, as well as environmental health indicators (4). In addition to their ecological services, reptiles have become desirable for food, medicinal products, pet trade, leather goods and research applications (5–7). However, their existence and well-being have constantly been threatened by several factors, such as hunting, environmental pollution, loss of habitat, destructive non-native species, climate change, and infectious diseases (4, 8–10). Disease surveillance and research in wild populations of reptiles are associated with numerous challenges including difficulties in accessing samples or field data, misleading epidemiological data and missing population data, as well as political and cultural restrictions (11). These challenges well explain the use of captive wildlife as models in many studies to acquire epidemiological information, since diseases are comparable in both wild and captive animals (12–15). Nonetheless, more robust and ideal epidemiological data are obtained when free ranging animals are surveyed. Recently, researchers have taken a renewed interest in reptilian viruses, partly due to the role played by reptiles as reservoir hosts for zoonotic viruses, as well as improvements in viral diagnostic methods that, in turn, have increased understanding of viruses in reptiles (16–20).

Herpesviruses (HVs) are members of the family *Herpesviridae*, a large taxon of DNA viruses that have been described in most vertebrate animals, including reptiles (16, 21). Herpesviruses are enveloped viruses with an icosahedral nucleocapsid and a linear double-stranded genome of varying length from ~124 to 259 kbp (22). Generally, HVs replicate within host cell nuclei and are able to remain latent in their natural hosts (17, 23). So far, reptilian HVs that have been identified and characterised all belong to the subfamily *Alphaherpesvirinae* (17, 24–26).

The occurrence of HV infections among reptiles has been widely documented and associated with stomatitis, tumors, encephalitis, conjunctivitis, hepatitis and mortalities (27, 28). Unfortunately, current treatment options of reptilian HVs are limited and the search for potent vaccines remains a herculean task; therefore, the adoption of preventative strategies is still the most efficient way of controlling these diseases. This review aims to assist in biosecurity planning as well as create a knowledge platform for decision makers and researchers by providing an overview of the taxonomy, pathogenesis, pathology and epidemiology of reptilian HVs.

## Methods

**Figure 1:**
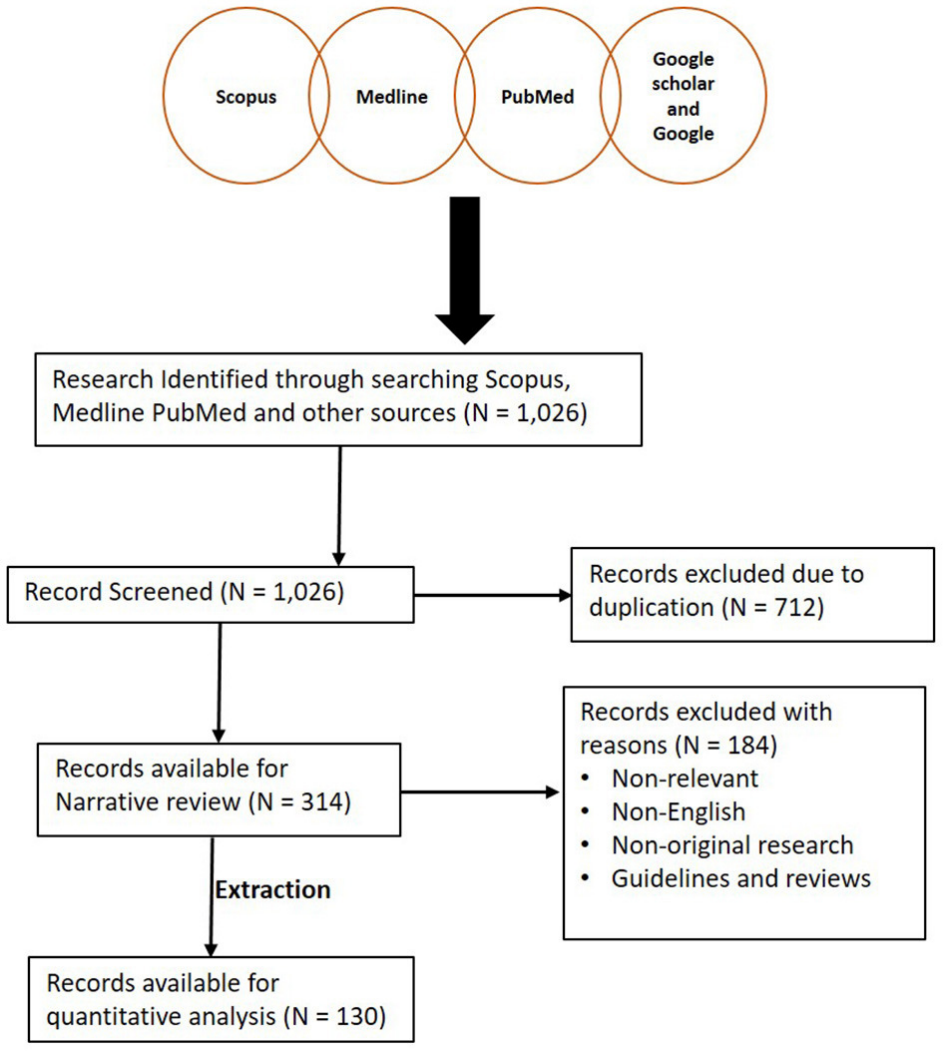
Summary of literature search conducted. A total of 1,026 articles were initially screened and 712 articles were later excluded due to duplication. A total of 314 articles were then reviewed for this study. One hundred and thirty studies were then extracted for quantitative analysis.

**Figure 2:**
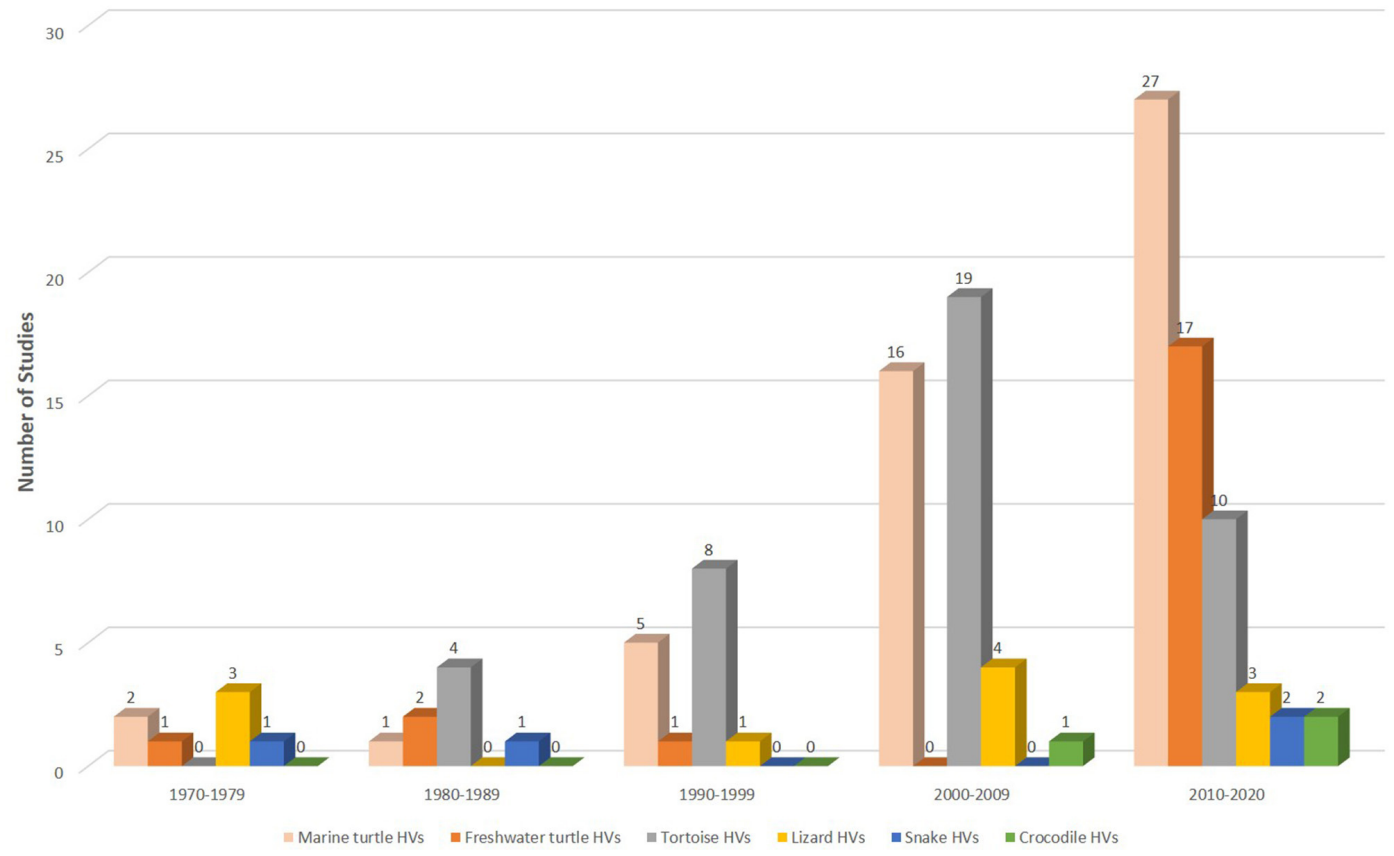
Studies on HVs in reptiles from 1972 to September 8, 2020. Overall, more than a quarter (*n* = 51; 39%) of the 130 extracted studies were conducted in marine turtles since the 1970s. Of the 60 publications between 2010 and 2020, 45% (*n* = 27) were ChHV-5 related studies.

**Figure 3:**
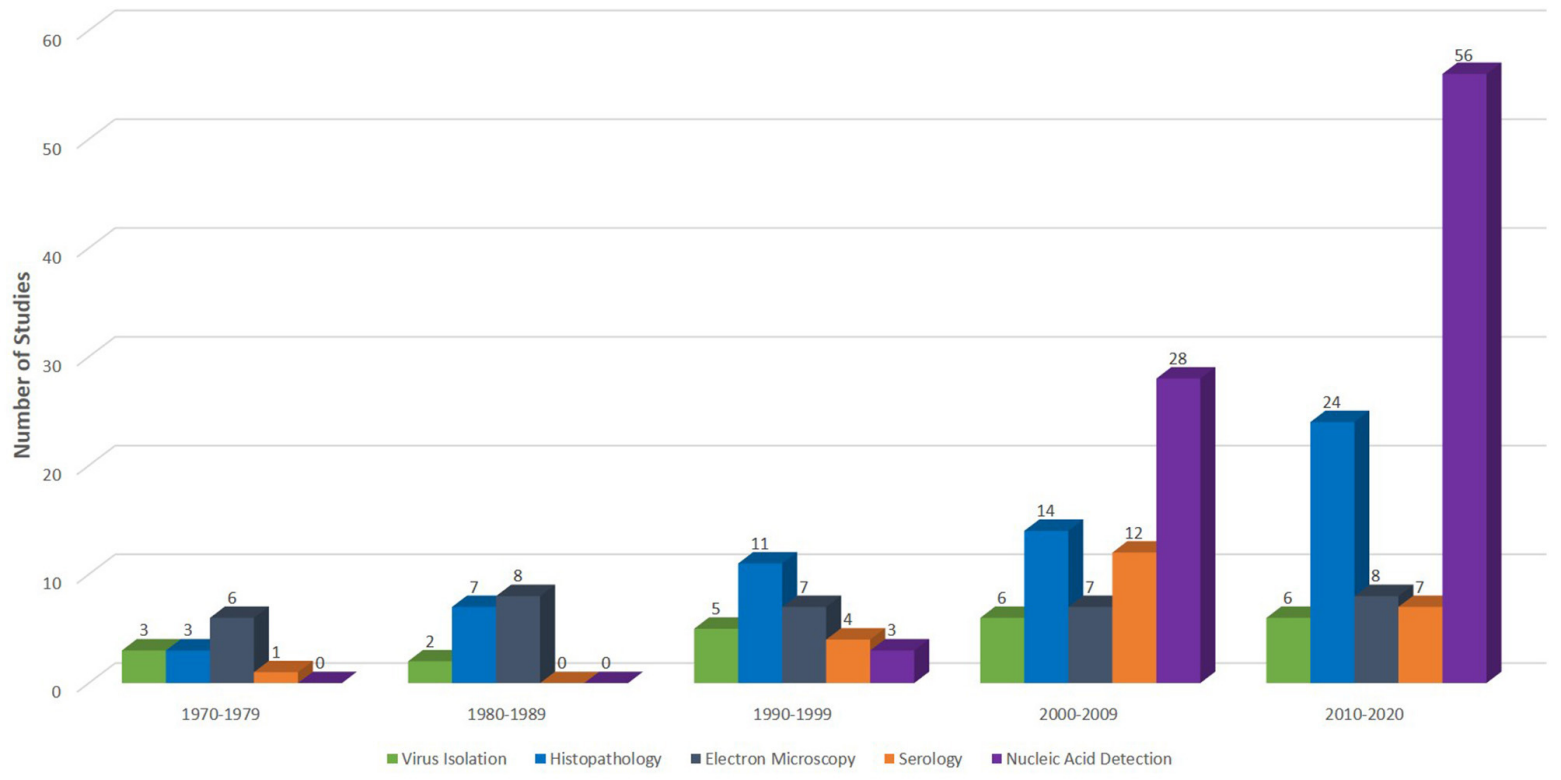
Diagnostic methods used in different studies from inception 1970 to September 8, 2020. Of the 130 studies extracted for this quantitative analysis, 22 (17%), 59 (45%), 36 (28%), 24 (18%), and 87 (67%) used virus isolation, histopathology, electron microscopy, serology, and nucleic acid detection assays, respectively, for various investigations of reptilian HVs.

Databases such as Medline (Ovid), PubMed, and Scopus were searched using specific keywords and phrases including *Herpesviridae* infections, herpesvirus infection, fibropapillomatosis, grey-patch disease, loggerhead genital-respiratory herpesvirus, herpesvirus disease, reptiles, turtles, tortoise, snakes, lizards, alligators, and crocodiles (Supplementary File 1). To ensure that relevant publications were not missed, each subheading was searched independently on PubMed. Also, an additional literature search was conducted by assessing references of articles selected from previous databases. A summary of the search results is shown in Figure 1. Furthermore, we read the abstracts and full texts of the selected articles, extracted and analysed information on the diagnostic methods used and the reptilian HVs investigated from 1972 to September 8, 2020 (Figures 2, 3; Supplementary File 2). Non-English, non-original research, guidelines, and review articles were excluded from the analysis.

## Bibliometrics

**Figure 4:**
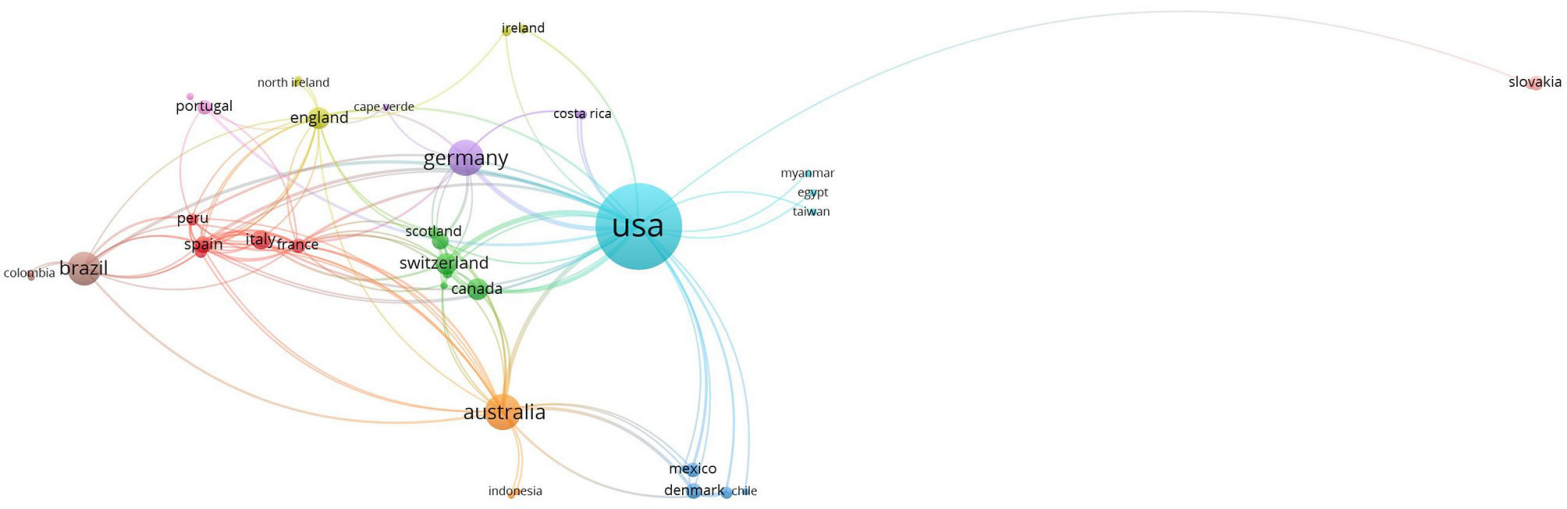
Links between collaborating authors from different countries with research output from inception to 2020. The circular coloured nodes represent countries and the node size indicates number of publications from the country. The lines between nodes indicate authorship collaborations between countries and the widths of these lines indicate the link strength.

We conducted bibliometric analyses of published articles on the topic of reptilian HVs using Vosviewer software (29) and the Web of Science Core Collection database. A total of 245 publications were downloaded from Web of Science Core Collection database using the following search terms; herpesvirus, turtle, lizard, snake, tortoise and crocodile. The strategy involved a combined use of the keywords, tags and Boolean operators to create query sets as follows: ALL= (herpesvirus) AND ALL= (turtle* OR lizard* OR snake* OR tortoise* OR crocodile*) with no limitations. USA had the highest number of research outputs with 149 (60.8%) articles. This was followed by Germany (28; 11.4%) and Australia (26; 10.6%) (Figure 4; Table 1). Using Vosviewer, we visualised the major keywords commonly used in the field of reptilian HVs and the link strengths between collaborating countries (Figure 4; Supplementary File 3). Of note, a low number of records on reptilian HVs were observed for some countries (Indonesia, Mexico and India) that have rich reptile diversity (Table 1) (3, 30, 31). Some of these countries also had little or no collaborations with the high research output countries (Figure 4), thus suggesting an under-reporting of reptilian HVs in these countries. Conversely, Germany has less reptile diversity with a higher number of records (Table 1). This observation could be attributed to the presence of established diagnostic resources or increased monitoring and reporting systems for reptilian diseases in the country.

**Table 1:** Bibliographic data on reptilian herpesviruses based on the number of articles from different countries.

**Countries/Regions**	**Records**	**% of 245**	**No. of reptiles species (3,31)**	^*^Rank
USA	149	60.8	1,147	2
Germany	28	11.4	29	31
Australia	26	10.6	1,159	1
Brazil	23	9.4	878	5
Switzerland	11	4.5	27	32
Italy	10	4.1	65	24
Canada	9	3.7	57	25
United Kingdom	15	6.1	6	43
Belgium	6	2.4	11	41
Spain	6	2.4	78	23
Denmark	5	2.0	12	40
France	4	1.6	181	15
Mexico	4	1.6	1,021	3
Portugal	4	1.6	44	27
Slovakia	4	1.6	16	37
Ecuador	3	1.2	541	10
Japan	3	1.2	111	22
China	3	1.2	605	8
Peru	3	1.2	586	9
Austria	2	0.8	18	36
Costa Rica	2	0.8	467	12
Czech Republic	2	0.8	15	38
India	2	0.8	889	4
Ireland	2	0.8	21	35
Netherlands	2	0.8	23	34
Barbados	1	0.4	13	39
Cape Verde	1	0.4	51	26
Chile	1	0.4	179	16
Colombia	1	0.4	654	7
Croatia	1	0.4	42	28
Egypt	1	0.4	133	20
French Guiana	1	0.4	177	17
Indonesia	1	0.4	798	6
Israel	1	0.4	148	19
Myanmar	1	0.4	374	13
Nicaragua	1	0.4	218	14
Norway	1	0.4	10	42
Romania	1	0.4	31	30
Seychelles	1	0.4	37	29
South Africa	1	0.4	529	11
South Korea	1	0.4	26	33
Taiwan	1	0.4	123	21
Turkey	1	0.4	150	18
Turks Caicos	1	0.4	12	40

* *Ranking was conducted based on the number of reptile species by countries identified from our bibliometric search and not based on the global ranking by Butler (31)*.

## Taxonomy of Reptilian Herpesviruses

Reptilian HVs belong to the family *Herpesviridae*, a member of the order *Herpesvirales* (32). According to the 2019 *International Committee on Taxonomy of Viruses* (ICTV) classification, the subfamily *Alphaherpesvirinae* comprises five genera namely, *IItovirus, Mardivirus, Scutavirus, Simplexvirus*, and *Varicellovirus*. Only the genus *Scutavirus* contains species that cause HV diseases in reptiles and includes *Chelonid alphaherpesvirus 5* (ChHV-5) and *Testudinid alphaherpesvirus 3* (TeHV-3). The species *Chelonid alphaherpesvirus 6* (ChHV-6) belongs to the subfamily *Alphaherpesvirinae* with unknown generic placement. The *Iguanid herpesvirus 2* (IgHV-2) that causes cytopathic infection in iguanids is of unknown generic and subfamilial placement (32–35).

**Figure 5:**
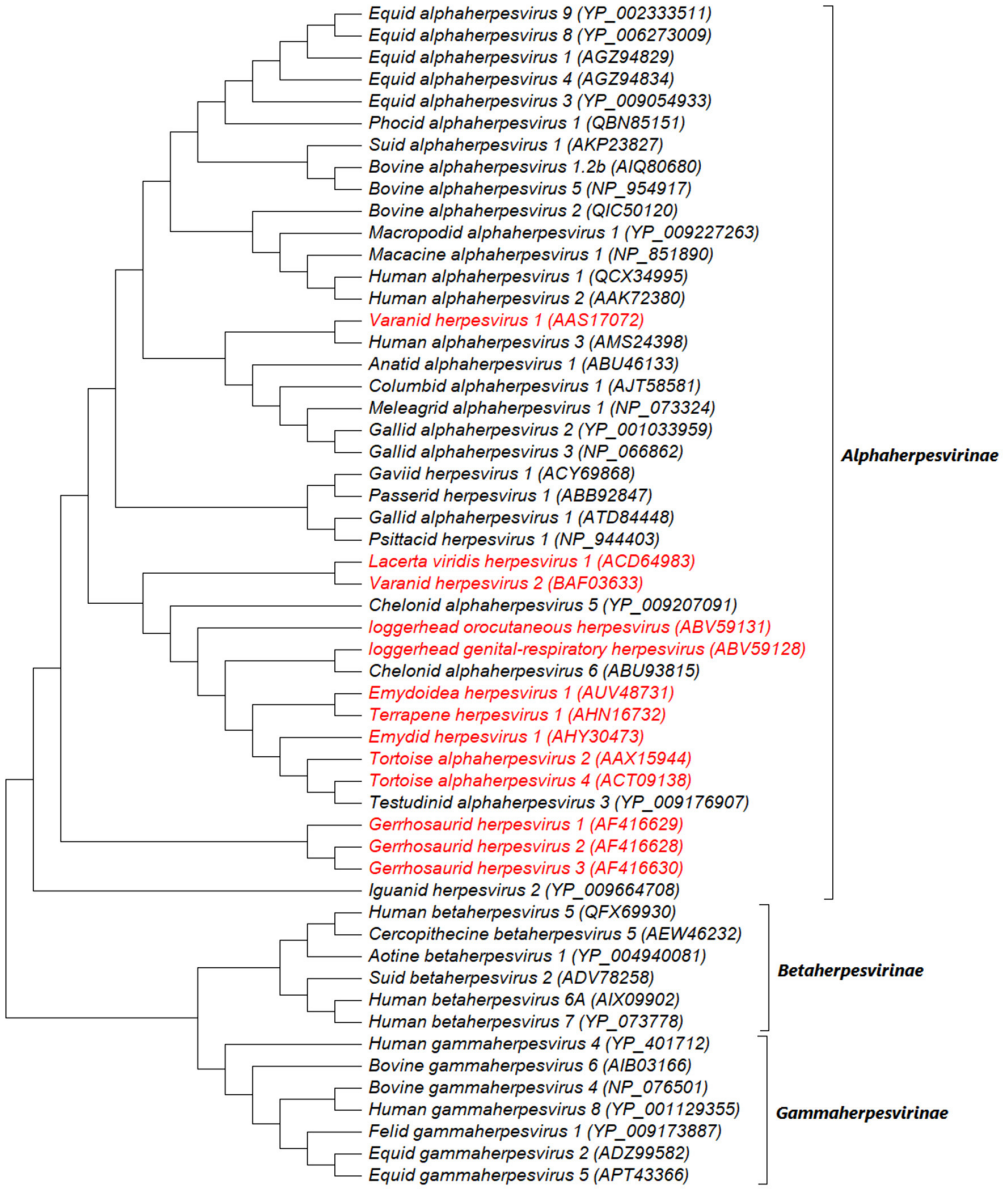
Midpoint rooted maximum likelihood phylogenetic tree of predicted amino acid sequences of HV-DNA-dependent DNA polymerase. The unassigned reptilian HVs are shown in red and cluster within the subfamily *Alphaherpesvirinae*. This analysis involved 54 amino acid sequences (37 complete and 17 partial sequences) downloaded from NCBI website (https://www.ncbi.nlm.nih.gov/) and aligned by ClustalW. There were a total of 1,443 positions in the final dataset. The evolutionary history was inferred by using the Maximum Likelihood method and JTT matrix-based model. The tree with the highest log likelihood (−60139.09) is shown. Initial tree(s) for the heuristic search were obtained automatically by applying Neighbor-Join and BioNJ algorithms to a matrix of pairwise distances estimated using a JTT model, and then selecting the topology with superior log likelihood value. The tree is drawn to scale, with branch lengths measured in the number of substitutions per site. Evolutionary analyses were conducted in MEGA X (59).

Since the end of the twentieth century, advances in molecular and phylogenetic analyses have made it possible for novel reptilian HVs to be identified with proposed taxonomic placements (36). Novel HVs have been detected in freshwater turtles, including Emydoidea herpesvirus 1 (EBHV-1), Pelomedusid herpesvirus 1, Glyptemys herpesvirus 1 and 2 (GlyHV-1 and−2), Emydid herpesvirus 1 and 2 (EmyHV-1 and−2), and Terrapene herpesvirus 1 and 2 (TerHV-1 and−2) (28, 37–41). Loggerhead genital-respiratory herpesvirus (LGRV) and loggerhead orocutaneous herpesvirus (LOCV) were detected in loggerhead turtles (*Caretta caretta*) and the genus *Chelonivirus* was proposed for these viruses and other related chelonian HVs (42). Also, tortoise HV species (TeHV-1,−2,−3,−4) have been identified and placed in the proposed genus Chelonivirus (43–46), although TeHV-3 has been formally assigned to the genus *Scutavirus* (32). Other unassigned reptilian HVs in the family *Herpesviridae* include the Iguanid herpesvirus 1 (IgHV-1), Gerrhosaurid herpesvirus 1-3, Varanid herpesvirus (VHV-1,−2,−3) and Helodermatid herpesvirus 1 (HeHV-1) in lizard species (25, 47–51), Opheodrys herpesvirus 1 in snakes (52), Crocodyline herpesvirus 1-3 (CrHV-1,−2,−3) in crocodiles (53), and, Chelonid herpesvirus 1-4 (ChHV-1,−2,−3,−4) in green turtles (*Chelonia mydas*-ChHV-1) (54), freshwater turtles (*Clemmys marmorata*-ChHV-2; *Chrysemis picta*-ChHV-3) (55, 56), and Argentine tortoise (*Geochelone chilensis*-ChHV-4) (57). Notably, some of these unassigned HVs were identified decades ago based on their morphological and biological characteristics using techniques (virus isolation, electron microscopy and histopathology) that were available at that time, thus making it challenging to place them taxonomically. We conducted a phylogenetic analysis to illustrate the relationship between the unassigned reptilian HVs and currently assigned HVs using amino acid sequences of HV-DNA-dependent DNA polymerase (37 complete and 17 partial sequences) from the NCBI website (https://www.ncbi.nlm.nih.gov/). As previously described (37, 58), the analysis showed that the unassigned reptilian HVs form a monophyletic group with members of the subfamily *Alphaherpesvirinae*. Freshwater HVs showed a close phylogenetic relationship with the tortoise HVs while the lizard HVs indicated high variations (Figure 5). Overall, given the variations shown by the unassigned reptilian HVs, it remains a matter of scientific deliberation whether these viruses should be assigned into one genus such as TeHV-3 and ChHV-5 or into different genera, although we envisage the latter would be the case.

## Virion and Genome Organization

All members of the family *Herpesviridae* share a common virion architecture, comprising a monopartite, linear, double stranded DNA core enclosed within an icosahedral capsid with a *T* = 16 symmetry (60–62). The capsid is tightly wrapped by a proteinaceous tegument, which, in turn, is surrounded by an envelope containing polyamines, lipids, and essential antigenic glycoproteins (62, 63). Unlike reptilian HVs, the atomic structures of human alphaherpesviruses have mostly been described. For instance, a cryo-electron microscopy (Cryo-EM) resolved the atomic structure of human simplexviruses (HSV-1 and−2), which comprise capsid organisation (hexons, pentons and triplexes), capsid proteins (VP5, VP19C, VP23, and VP26) and tegument proteins (pUL17, pUL25, and pUL36) (64–66). Although the atomic structures of reptilian HVs have not specifically been resolved, the resolved structures of other alphaherpesviruses provide insights, since their genomes have many similarities (36, 67). Consequently, the insights could guide future research in the atomic structure resolutions of reptilian HVs, which in turn could serve as a baseline for reptilian HV vaccinology.

Although the complete nucleotide sequences for most reptilian HVs are yet to be obtained, genomic features can be inferred from other fully sequenced alphaherpesviruses owing to sequence homology (36, 67). All alphaherpesvirus genome structures contain unique long (U_L_) and short (U_S_) sequences and each are flanked by both terminal (TR_L_, TR_S_) and internal (IR_L_, IR_S_,) inverted repeat regions, giving the general configuration TR_L_-U_L_-IR_L_-IR_S_-U_S_-TR_S_ (68). The complete genome of two TeHV-3 strains (1976 and 4295) was recently sequenced. The 1976 strain was shown to have a novel inverted repeat (TR_T_, IR_T_) and unique (U_T_) regions (69). The genome is approximately 160 kbp, encodes at least 107 open reading frames (ORFs) and consists of U_L_ (107,928 bp) and U_S_ (20,375 bp) regions. The U_L_ is bound to its right by the U_S_ adjoined to inverted repeats (IR_S_ and TR_S_; 8,536 bp) and to its left by a third unique region (U_T_; 12,595 bp), which is also bordered by inverted repeats (TR_T_ and IR_T_; 1,194 bp) to give the overall layout TR_T_-U_T_-IR_T_-U_L_-IR_S_-U_S_-TR_S_ (69). However, this differs from the type D configuration earlier attributed to this species (70). In another study, the complete nucleotide sequence of a Bacterial Artificial Chromosome (BAC) containing the entire genome of ChHV-5 (cloned in pTARBAC2.1) was obtained and it showed a different configuration (U_L_-IR_S_-U_S_-TR_S_) from that of TeHV-3, even though they both belong to the genus *Scutavirus* (71). Moreover, the genome characterisation of strain 4295 identified regions containing genes that could be involved in viral pathogenesis or virulence (69). This is an important finding as these regions could serve as therapeutic or diagnostic targets in future research. Similarly, evidence of recombination among strains of ChHV-5 documented by Morrison et al. could lead to increased virulence and transmission of ChHV-5 variants (72) and these events may remain undetected in sea turtle populations. Therefore, it is pertinent to strengthen current diagnostic approaches to allow for more comprehensive geographical surveys and characterisation of HVs. Also, as new and affordable diagnostic techniques are being developed and improved upon, we expect more novel structures of reptilian HVs to be reported.

## Transmission and Pathogenesis

Several modes of reptilian HV transmission have been postulated including vertical, horizontal and mechanical transmissions (73–75). Marenzoni et al. reported the first evidence of vertical transmission of TeHV-3 in a captive breeding facility (14). In this study, one hatchling born in isolation from the egg laid by an infected tortoise (*Testudo hermanni hermanni*) presented with conjunctivitis and tested positive by specific polymerase chain reaction (PCR) targeting the partial sequence of the UL39 gene of TeHV-3 (14). In other studies, Jones and colleagues provided molecular evidence for the horizontal transmission of ChHV-5 in green turtles by demonstrating the molecular link between viral variants and foraging grounds (76, 77). Furthermore, experimental studies have revealed the possible transmission of reptilian HVs by direct contact with infectious secretions (78–80) or indirectly via vectors and water (81–84). By linking viral shedding patterns or frequency to disease occurrence, we could trace the most probable transmission mode of reptilian HVs. For instance, in captive enclosures, HVs could be easily transmitted via contact with secretions or contaminated materials, even at low shedding rates, and the removal of infected animals and infectious materials could stop the spread of the virus. In the wild, different transmission agents such as vectors, fomites and superspreaders could interplay to compensate for the low contact rate and infrequent shedding of some HVs (85). Because of the managerial implications, it is therefore important to further investigate the roles of these factors in the transmission of HVs in wild reptiles.

Four pathogenic mechanisms are highly conserved among all HVs and include: (1) intranuclear replication and capsid assembly; (2) expression of DNA metabolic and synthetic enzymes; (3) cell destruction following the release of viral progenies; and (4) the maintenance of latency in natural host cells (63, 86, 87). Generally, HV infections begin with viral entry, which is followed either by localisation or systemic spread (87). An experimental transmission study described the systemic dissemination of tortoise HVs (isolates HV 1976 and HV 4295/7R/95) (78). Following experimental infection via intranasal and intramuscular routes, the HVs elicited clinical signs and were detected by PCR in tissue samples from the respiratory, digestive and urogenital tracts, central nervous system (CNS), heart and spleen (78). Fibropapillomatosis (FP) and grey patch disease (GPD) are both associated with clinical signs that could be attributed to local destruction of infected cells due to replication and progeny release (54, 80, 88–90). Evidentially, two studies demonstrated the local replication of ChHV-5 by detecting certain biomarkers (eosinophilic intranuclear inclusions, F-VP26, DNA, and mRNA transcripts) within fibropapillomatous lesions (85, 91).

Unfortunately, the specific mechanisms involved in host cell invasion, immune evasion, localisation and spread of reptilian HVs have not been fully elucidated. However, recent molecular studies have provided insights into some virulence factors associated with reptilian HVs (69–71). Briefly, glycoproteins B (gB), gC, gD, gH, gK gL, gM, and gN have been hypothesised to function in host cell attachment and entry (69–71). gB and gC are capable of binding to heparan sulphate proteoglycans that are present on the surface of many cells, thus aiding viral adsorption and penetration into different cell types (69, 92). The interactions of gB, gD, gH, gK, gL, gM, and gN have also been postulated to mediate membrane fusion and viral entry into the cell (69, 70, 92). Glycoproteins B, E, H, and L are involved in viral cell to cell spread, which could occur through intercellular bridges or intra-axonal transport, thus circumventing humoral immune responses (69, 70). Similarly, the gC can bind to the third complement component (C3b) to block the alternative pathway complement activation (69, 93). The gE and gI in HSV-1 inhibit the normal function of antibodies by building up a complex that acts as an Fc-receptor (94). However, the immunosuppression mechanism of the gE homologue in reptilian HVs is not yet clear. Finally, the F-M04 and F-sial proteins were recently identified in ChHV-5 and thought to play a role in FP pathogenesis; however, the specific mechanisms involved are not yet understood (71). Future research should consolidate characterisation of reptilian HVs in order to increase the understanding of host-pathogen interactions and improve clinical interventions.

## Clinical and Pathological Signs

**Figure 6:**
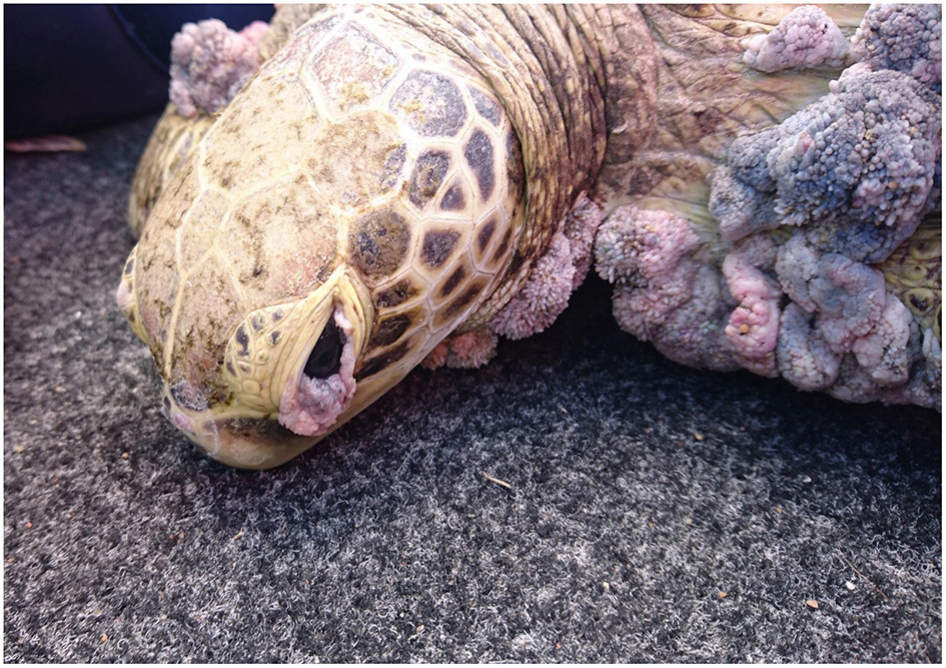
Fibropapillomatosis in green turtle (*Chelonia mydas*). Photo by Dr. Karina Jones.

**Figure 7:**
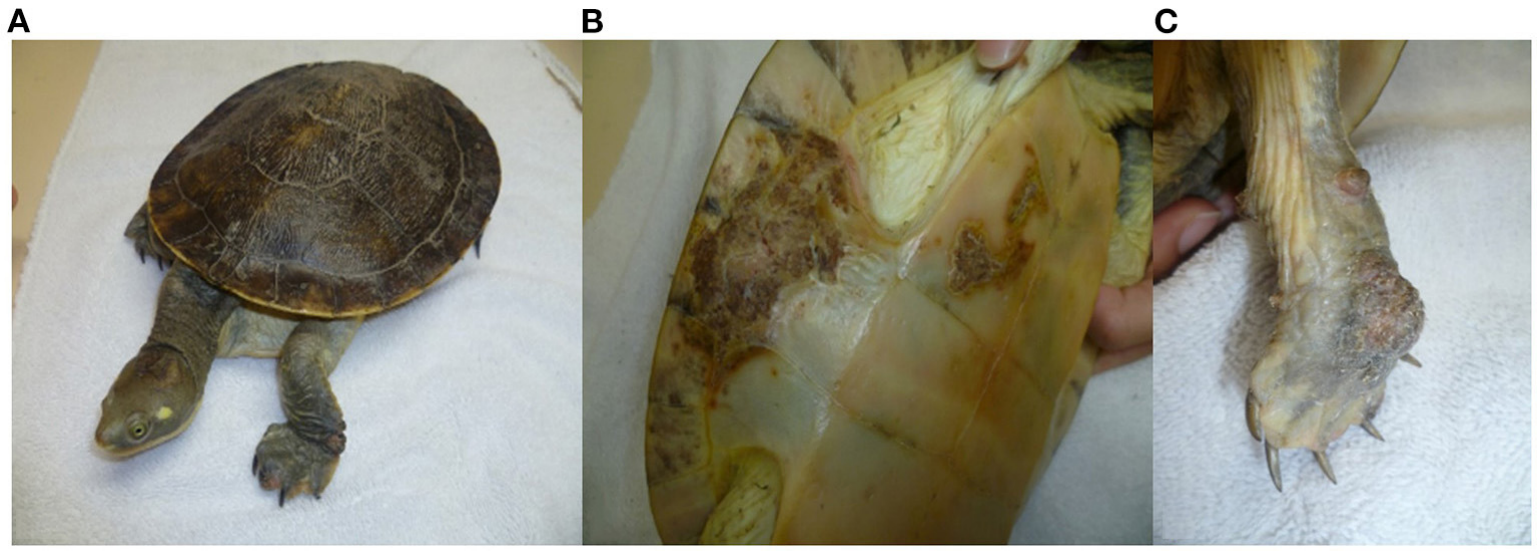
Herpesvirus infection in freshwater turtle (*Emydura macquarii krefftii*) presented with proliferative and ulcerative lesions of the skin **(A)**, proliferative and crusted lesions on the bridge of the shell **(B)**, and proliferative lesion on the palmar aspect of the right forefoot **(C)**. “Adapted from Herpesvirus in a captive Australian Krefft's river turtle (*Emydura macquarii krefftii*)” by Cowan et al. (110). Copyright 2021 by John Wiley and Sons. Reprinted with permission.

**Figure 8:**
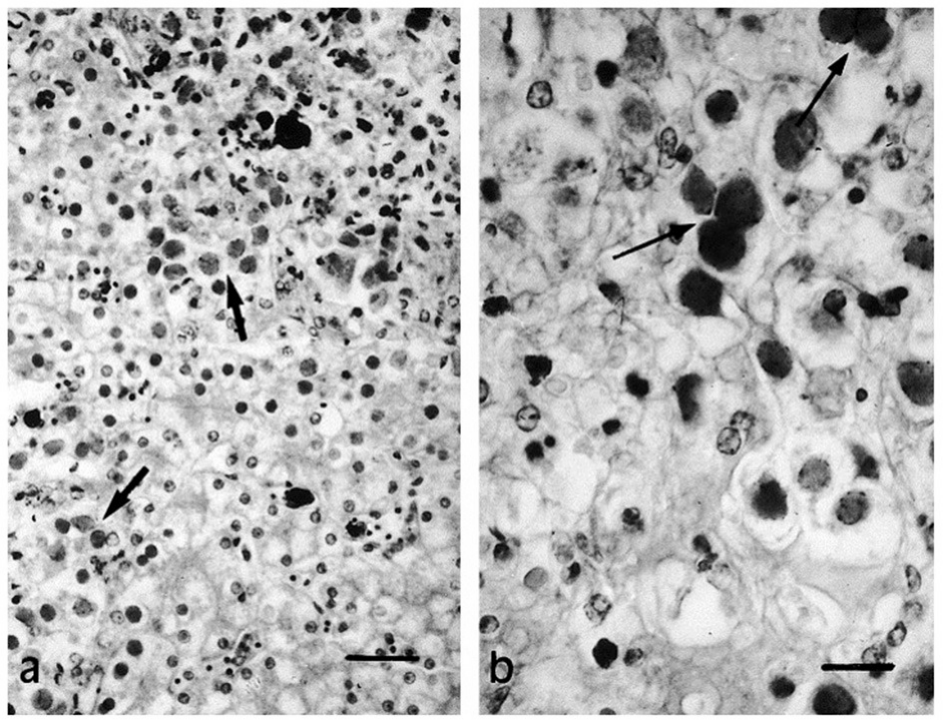
Necrotic foci **(a)** and syncytial formation **(b)** in HV infected hepatocytes of a tortoise (*Testudo horsfieldii*). “Adapted from Hepatitis Associated with Herpes Viral Infection in the Tortoise (*Testudo horsfieldii*)” by Hervás et al. (112). Copyright 2021 by John Wiley and Sons. Reprinted with permission.

Herpesvirus infections have been described in reptiles with a range of clinical manifestations (16). To provide an overview, the clinical signs and the gross and histological lesions associated with reptilian HVs are summarised in Table 2. Some of the more detailed descriptions are from sea turtles, tortoises and crocodiles. Grey patch disease and FP, characterised by coalescing greyish papular skin lesions (spreading patches) and branching papillary tumours (Figure 6), respectively, have been reported in sea turtles (42, 54, 108, 109). Lung-eye-trachea disease (LETD) with a clinical course of 2–3 weeks has been seen in green sea turtles (98). Lung-eye-trachea diseased turtles often present with pneumonia, stridor and caseation of the eyes, oropharynx and trachea (98). In freshwater turtles, HV infections are associated with hepatic necrosis, and proliferative and/or ulcerative lesions of the skin and shell (Figure 7) (37, 38, 110, 111). Infections in tortoises result in ulcerative to diphtheroid-necrotizing stomatitis, conjunctivitis, glossitis, rhinitis, dyspnoea, liver disease and neurological disease and could be accompanied by anorexia, regurgitation, neck oedema, lethargy and death (Figure 8) (112–115). Papillomas, stomatitis, and hepatitis are commonly described in lizards infected with HV (25, 47–49, 116, 117). Recently, five green snakes (*Opheodrys vernalis*) housed together presented with oropharyngeal squamous cell carcinoma and molecular analysis confirmed the presence of a novel Opheodrys herpesvirus-1 (*Alphaherpesvirinae*) (52). In another study, a lymphoid follicular cloacal inflammation in juvenile alligators was initially associated with tortoise HV. However, the HV (Genbank accession AY913769.1) was later determined to be a likely laboratory contamination and the actual causative agent is still unknown (118). Similarly, Hyndman et al. identified three novel HVs associated with conjunctivitis and/or pharyngitis (CP), systemic lymphoid proliferation with non-suppurative encephalitis (SLPE), and lymphonodular skin lesions (LNS) in farmed saltwater crocodiles (*Crocodylus porosus*) and captive freshwater crocodiles (*Crocodylus johnstoni*) (53). Obviously, HVs can induce significant diseases in both captive and wild reptiles; therefore, there is a need to develop rapid diagnostic tests that will aid disease surveillance and reporting in order to maintain safe biosecurity measures and reduce spread.

**Table 2:** Clinical presentations, gross, and histological lesions of reptilian HV species.

**Species**	**Reported host**	**Clinical presentation**	**Gross lesion**	**Histopathology**	**References**
ChHV-1	• Green sea turtles (*Chelonia mydas*)	• Benign papular lesions on the neck and flippers • Spreading grayish patches to large areas of the epidermal surface • Death may occur	• Benign Papules • Spreading gray patches	• Intranuclear inclusions found in epidermal keratinocytes	(54, 95, 96)
ChHV-2	• Pacific pond turtles *(Clemmys marmorata*)	• Lethargy • Anorexia • Muscular weakness • Coma • Subcutaneous haemorrhages • Death	• Hepatomegaly • Pallor of kidney • Subcutaneous Petechial and ecchymotic haemorrhages	• Hepatic necrosis • Intranuclear inclusion bodies • Lymphocytic aggregation in liver, kidney, and spleen • Moderately hyperplastic spleen	(55)
ChHV-3	• Painted turtles (*Chrysemys picta*)	• Abscessation • Death	• Pulmonary edema • Friable and greenish-brown liver • Distended gall bladder • Congested spleen • Shell rot lesions on plastron	• Foci of necrosis on the liver and infundibular septa • Hepatocytes containing Eosinophilic intranuclear inclusions • Granulocytic and mononuclear infiltrations	(56)
ChHV-4	• Argentine tortoise (*Geochelone chilensis*)	• Acute death • Nasal discharge • Ocular discharge • Regurgitation • Anorexia • Lethargy • Necrotizing stomatitis	• Necrotizing lesions • Serous atrophy of fat • Pale liver	• Diffuse area of necrosis in mucosal epithelium • Accumulation of necrotic cellular debris and fibrin • Infiltration of Inflammatory cells • Eosinophilic intranuclear inclusions within degenerating epithelial cells and other tissues • Vacuolar degeneration of hepatocytes	(57)
ChHV-5	• Green sea turtle (*Chelonia mydas*) • Loggerhead sea turtle (*Caretta caretta*) • Hawksbill turtle (*Eretmochelys imbricata*) • Leatherback turtle (*Dermochelys coriacea*) • Olive ridley sea turtle (*Lepidochelys olivacea*) • Kemp's ridley sea turtle (*Lepidochelys kempii*) • Flatback turtle (*Natator depressus*)	• Tumours on the inguine, tail, flippers, axillae, chin, neck, eyelids, corneas, carapace and plastron	• Single to multiple raised cutaneous masses that are verrucous, smooth, sessile or pedunculated • Ulcerated and necrotic large masses • Pigmented cutaneous tumours • Spherical, smooth, firm, white, or gelatinous and translucent nodules in the lungs, kidneys, liver, heart, and gastrointestinal tract	• Papillary epidermal and dermal hyperplasia • Orthokeratotic hyperkeratosis • Hypertrophied epithelial cells overlying vascularized fibrous stroma • Epithelial necrosis and multifocal areas of ballooning degeneration • Lymphocytes and plasma cells infiltrations • Melanophores within the masses • Eosinophilic intranuclear inclusions	(97)
ChHV-6	• Green sea turtles (*Chelonia mydas*)	• Gasping • Buoyancy abnormalities • Inability to dive • Lethargy • Caseous exudate covering the eyes, glottis and trachea • Death	• Emphysematous areas in the lungs • Caseous exudate in the eyes, glottis and trachea • Multifocal raised white nodules in the liver	• Necrotic lesions in the glottis, tracheal and lungs • Periglottal accumulations of necrotic cellular debris and fibrin • Infiltrations of heterophils, lymphocytes, and plasma cells in periglottal submucosa • Periglottal and tracheal epithelial proliferative and/or squamous metaplastic changes • Syncytial giant cells in tracheal mucosa and major airways of lungs • Thickened interstitium	(98)
				• Hypertrophic and hyperplastic cells with enlarged vacuolated nuclei lining the airways • Amphophilic intranuclear inclusions	
TeHV-1	• Horsfield tortoises (*Testudo horsfieldii*) • Pancake tortoises (*Malacochersus tornieri*)	• Cervical extension • Laboured breathing • Respiratory murmur • Oral and nasal discharge • Reddish-white fibrinous coating of the tongue • Death	• Yellowish-white pseudomembrane in the mouth, pharynx and glottis • Hepatomegaly and ecchymotic liver • Pseudomembrane formation in the stomach	• Diffuse areas of degeneration and necrosis in tongue and pharynx and larynx • Necrotic cellular debris and fibrin accumulation • Inflammatory cells infiltrations • Eosinophilic or amphophilic inclusion bodies	(99–101)
TeHV-2	• Desert tortoises (*Gopherus agassizii*)	• Anorexia • Lethargy • Necrotizing stomatitis	• Oral plaques	• Eosinophilic and amphophilic intranuclear inclusions in superficial epithelial cells • Thick coagulum over the epithelial surfaces of the mouth, pharynx, and trachea • Infiltration of heterophils, lymphocytes, plasma cells, and macrophages • Granulation of oropharyngeal tissue following epithelial loss	(44)
TeHV-3	• Greek Tortoises (*Testudo graeca*) • Hermann's Tortoises (*Testudo hermanni*)	• Nasal and oral discharges • Rhinitis • Dyspnoea • Conjunctivitis associated with blepharospasm • Diphtheroid-necrotizing stomatitis • Glossitis • Pharyngitis • CNS involvement (Circling, head tilt, lethargy, circling, paralysis and incoordination) • Deaths	• Stomatitis with yellowish oral plaques • Rhinitis with foamy nasal discharge • Conjunctivitis	• Oesophageal hyperplasia • Hyperplasia and hyperkeratosis in the oral mucosa • Sloughing of the epithelial cells and multifocal erosion • Glottal epithelial ulceration, hyperplasia and necrosis • Heterophilic pustules. • Amphophilic intranuclear inclusion bodies • Heterophilic bronchitis and pneumonia • Nuclear degeneration changes of the hepatocytes • Ballooning degeneration renal and digestive organs	(14, 78, 102, 103)
TeHV-4^*^	• Bowsprit tortoise (*Chersina angulata*) • Leopard tortoise (*Stigmochelys pardalis*)	• Asymptomatic in some cases. • Respiratory distress • Increased salivation	• No data	• No data	(43, 104)
LGRV	• Loggerhead sea turtles (*Caretta caretta*)	• Moribund state • Lethargy and quadriparesis • Emaciation • Abnormal gait • Death	• Colon impaction • Fibrinonecrotic colitis • Linear ulcers around the base of the base of the phallus • Multifocal ulcers along the mucocutaneous junction of the eyelids • Circumferential ulcer around the entire mucocutaneous junction of the cloaca • Ulcerative gastritis	• Epithelial hyperplasia. • Ballooning degeneration and syncytial cell formation within basal layers of the epithelium • Intranuclear eosinophilic inclusion bodies • Heterophilic inflammation	
LOCV	• Loggerhead sea turtles (*Caretta caretta*)	• Moribund state • Lethargy, bradycardia, hypoventilation, and aspiration pneumonia. • Death	• Deep multifocal ulcers around the rostral aspect of the tongue • Multifocal pale cutaneous plaques with erythematous borders on the ventral neck region • Tenacious exudates covering some plaques • Liver pallor	• Eosinophilic intranuclear inclusion bodies • Necrosis of the epithelium and extend into the underlying lingual collagen • Heterophilic inflammation • Epidermal hyperplasia • Hyperkeratosis • Intraepithelial pustules • Multifocal serocellular crust • Sloughed epithelial cells in the airways	(42)
EBHV-1^*^	• Blanding's turtles (*Emydoidea blandingii*)	Asymptomatic	• No data	• No data	(39)
GlyHV-1^*^	• Bog turtles (*Glyptemys muhlenbergii*)	Asymptomatic	• No data	• No data	(37)
GlyHV-2^*^	• Wood turtles (*Glyptemys insculpta*)	Asymptomatic	• No data	• No data	(37)
EmyHV-1	• Eastern river cooter (*Pseudemys concinna*) • Northern map turtle (*Graptemys geographica*) • Painted turtles (*Chrysemys picta*)	• Weakness • Frothy nasal discharge • Acute death	• Dark red, wet, and heavy lungs • Thickened, wet, and gelatinous cranial aspect of the lungs • Trace amount of watery fluid in the trachea • Diffusely tan, and slightly rounded lobular edges of the liver	• Hepatic lipidosis • Intranuclear inclusion bodies • Necrotic lesions in the lungs, liver and spleen • Granulocytic and lymphocytic interstitial infiltrations • Acute congestion with multifocal haemorrhage	(28, 105)
EmyHV-2^*^	• Bog turtle (*Glyptemys muhlenbergii*) • Spotted turtles (*Clemmys guttata*)	Asymptomatic	• No data	• No data	(37)
TerHV-1^*^	• Eastern box turtles (*Terrapene carolina carolina*)	• Lethargy • Dehydration • Dyspnoea • Moribund state with fibronecrotic stomatitis and cloacitis • Conjunctivitis • Blepharoedema • Death	• No data	• Necrosis, ulceration and syncytia formation of the pharyngeal mucosal epithelium • Eosinophilic to amphophilic intranuclear inclusions	(40)
TerHV-2	• Eastern box turtles (*Terrapene carolina carolina*)	• Papillomatous skin lesions • Anorexia	• Cutaneous papillomas	• Papillary hyperplasia of the epithelium • Infiltrations of lymphocytes, plasma cells, and heterophils • Epithelium covered by keratin and cell debris	(38)
Pelomedusid HV-1^*^	• West African mud turtles (*Pelusios castaneus*)	• Asymptomatic	• No data	• No data	(41)
IgHV-1	• Green iguana (*Iguana iguana*)	• Acute death	• Thin body • Generalized muscle wasting • Loss of fat store	• Hepatocellular necrosis • Hepatic syncytia • Eosinophilic intranuclear inclusions • Stomach and intestinal ulceration and necrosis • Acute renal tubular necrosis • Splenic lymphoid atrophy or hypoplasia	(33, 50, 51)
IgHV-2	• San Esteban Chuckwalla (*Sauromalus varius*)	• Acute death	• Haemorrhage in the lung • Congestion of airway • Pale liver	• Diffuse hepatic necrosis, eosinophilic intranuclear inclusions • Multifocal necrosis of the spleen • Interstitial infiltrations of muscles by mononuclear leucocyte • Fibrosis of muscle and gingiva	(34)
Gerrhosaurid HV-1	• Sudan plated lizard (*Gerrhosaurus major*)	• Glossal stomatitis • Severe dyspnoea	• Raised and tanned periglottal tongue • Little body fat	• Glottal trachea of granulocytic and lymphocytic inflammation with erosion of overlying epithelium	(47)
Gerrhosaurid HV-2^*^	• Black-lined plated lizard (*Gerrhosaurus nigrolineatus*)	• Labial stomatitis	• No data	• No data	(47)
Gerrhosaurid HV-3^*^	• Sudan plated lizard (*Gerrhosaurus major*)	• Chronic labial proliferative and ulcerative growth	• No data	• No data	(47)
VHV-1	• Green tree monitor lizards (*Varanus prasinus*)	• Proliferative and Ulcerative stomatitis/gingivitis • Squamous cell carcinomas	• Small white chalky plaques in the coelomic membrane, thoracic musculature, liver, kidneys, heart, and joints fascial plane • Gingival proliferation • Mucosal hyperplasia • Fibrinous exudate on the serosa of the gall bladder • Oral villous-like proliferation with patches of focal erythema	• Mucosal epithelial proliferation • Severe pulmonary, myocardial, hepatic, and renal vascular thrombosis • Sloughed tubular endothelial cells • Gingival necrosis • Hepatic lipidosis • Hepatic and renal amyloidosis	(48)
VHV-3	• Monitor Lizards (*Varanus spp*.)	• Acute death	• Yellow-tan or white viscous material and white, thick material in the intestine and distal colon, respectively • Multiple soft, white particles (2–3 mm) in intestinal tract • Diffuse pale-brown liver with multiple flat, tan pinpoint foci on the capsular surface	• Acute, multifocal, coagulative necrosis in the lamina propria of the small intestine • Acute, multifocal hepatocellular coagulative necrosis • Eosinophilic intranuclear inclusions in the small intestine and liver	(49)
HeHV-1	• Gila monster (*Heloderma suspectum*)	• Intraoral mass • Loss of weight	• Gingival nodule • Muscle atrophy	• Anastomosing epithelial cords • Proliferative gingival tissues • Eosinophilic and birefringent material within mass	(25)
Elapid HV-1	• Siamese cobra (*Naja naja kaouthia*)	• Thick tenacious venom (low grade venom)	• Enlarged venom gland • Thick venom exudates	• Venom glands are lined by degenerated epithelial cells • Mononuclear cell infiltration of gland subepithelium • Debris, degenerated cells and venom in the lumina of glands • Intranuclear inclusions	(106)
Opheodrys HV-1	• Smooth green snakes (*Opheodrys vernalis*)	• Oropharyngeal squamous cell carcinoma	• Pale tan, multinodular masses on oropharyngeal mucosa • Brown friable accumulations on tumour surface	• Distorted oropharyngeal mucosa and submucosa by epithelial neoplasm • Islands of neoplastic epithelial cells containing keratin cores • Anisocytosis and anisokaryosis of neoplastic epithelial cells	(52)
				• Squamous differentiation, keratin pearls, prominent intercellular bridges • Heterophilic inflammation and surface compact keratin layers	
CrHV-1	• Saltwater crocodiles (*Crocodylus porosus*)	• Conjunctivitis-pharyngitis (CP).	• Reddening and swelling of the conjunctivae of the eyelids and nictitating membrane • Cornea opacity and rupture • Fibrinocaseous conjunctival, lingual and oropharyngeal exudates	• Epithelial Hyperplasia, erosion, or ulceration of the conjunctiva, pharynx and larynx with cellular infiltrations • Lymphocyte, heterophil, and macrophage infiltrations of cornea, iris, and conjunctival, pharyngeal and laryngeal epithelium	(53, 107)
CrHV-2	• Saltwater crocodiles (*Crocodylus porosus*)	• Conjunctivitis-pharyngitis (CP) • Concurrent skin ulcers. • Systemic lymphoid proliferation and encephalitis (SLPE) • Lymphnodular skin (LNS)	**CP** • Gross lesions of CP syndrome as described above **SLPE** • Poor body condition • Splenomegaly. • Pulmonary edema **LNS** • Pale, soft, raised, well-delineated foci on lateral abdominal scales with occasional ulcerated surface covered in caseous exudate • Pale pink soft glistening tissue between the epidermis and deep dermal collagen • Enlarged tonsils with multinodular appearance • Discrete soft white foci in the subepithelial tissue of the conjunctiva • Multinodular swelling of the cloacal mucosa • Discrete white soft foci in the parenchyma of the myocardium, liver, or kidney	**CP** • Histological lesions of CP syndrome as described above **SLPE** • Lymphohistiocytic and macrophage infiltration of pulmonary septae, hepatic periportal regions, pancreatic interstitium, gastrointestinal submucosa, pericardium, epicardium, iris, wall of large blood vessels and brain • Hyperplastic lymphocytic conjunctivitis **LNS** • Expansion and displacement of collagen of the superficial and mid-dermis by intense multinodular mononuclear cell infiltrate • Epithelial hyperplasia of the tonsils will lymphocytes and macrophage infiltrations • Dense lymphohistiocytic aggregates of myocardium, liver, or kidney	(53, 107)
CrHV-3	Freshwater crocodiles (*Crocodylus johnstoni*)	• Systemic lymphoid proliferation	• Gross lesions of SLPE described above	• Histological lesions of SLPE described above	(53, 107)

*ChHV, Chelonid herpesvirus; TeHV, Testudinid herpesvirus; LGRV, loggerhead genital-respiratory herpesvirus; LOCV, loggerhead orocutaneous herpesvirus; EBHV, Emydoidea herpesvirus; GlyHV, Glyptemys herpesvirus; EmyHV, Emydid herpesvirus; TerHV, Terrapene herpesvirus; IgHV, Iguanid herpesvirus; VHV, Varanid herpesvirus; HeHV, Helodermatid herpesvirus; CrHV, Crocodyline herpesvirus*.

* *To the best of our knowledge, the gross or histological lesions of some novel viruses have either not been detected, or were reported while this manuscript was being written*.

## Epidemiology

Epidemiological studies of HVs in wild reptiles could be challenging due to a lack of sensitive diagnostics for the detection of unknown or novel species, especially in resource-limited regions. HV infections are commonly characterised by non-specific clinical signs in most reptiles, thus making diagnoses on the basis of clinical signs alone difficult. An exception to this is FP, in which the presence of cutaneous tumours gives an indication of the disease; hence, more FP-associated HV data have been reported in recent decades (Figure 2; Supplementary File 2). Even so, the complete disease impact on wild populations could be currently underestimated due to the underreporting of outbreaks, sampling bias and poor monitoring systems. For instance, there is a paucity of information for FP epidemiology at the pelagic phase of life in sea turtles as most studies are biassed towards sampling nearshore juveniles and adult females at foraging grounds or nesting beaches. Nevertheless, wildlife workers and researchers who, despite numerous challenges, have provided considerable epidemiological data targeted at conservation efforts towards endangered species should be commended. An overview of some of the epidemiological information including the prevalence and demography of both wild and captive reptilian HVs is discussed in this section.

Herpesviruses are linked to different diseases of marine turtles, including FP, LETD and GPD (54, 82). FP is a debilitating disease characterised by the development of tumours (119, 120). Depending on the location of the tumours, FP can have detrimental effects (109, 121). On the basis of prevalence and distribution, Tagliolatto et al. reported a prevalence rate of 43% for FP in green turtles captured in a foraging area in south-eastern Brazil (121). Adnyana et al. recorded 22% overall prevalence in green turtles in Indonesia and also observed that the prevalence rate of FP was higher among turtles from waters adjacent to densely populated regions compared to those collected from waters remote from urbanised regions of Indonesia (122). These findings indicate that the epidemiology of FP in marine turtles vary between geographical regions and may be linked to anthropogenic activity. This theory is supported by the findings in another study, which attributed the variation of FP prevalence to environmental cofactors that vary among local habitats (123). A study associated the geographical distribution of FP with the genomic variation of HVs in marine turtles, and observed four forms of the virus corresponding to Atlantic Ocean, west Pacific, mid-Pacific, and east Pacific (124). A similar study conducted in Australian waters identified different genotypes along the east coast of Queensland. Such differences in strains may also effectuate different levels of pathogenicity between strains (76, 77) and account for variation in reported prevalence in different regions. Also, given that the immune system of reptiles is dependent on temperature (125, 126), the variation in the prevalence rates of reptilian HVs across regions could be associated with differences in regional climate types. Comprehensive reviews of the epidemiology of FP in marine turtles have been documented elsewhere (73, 97, 127–129). The host immune status influences the clinical course of a disease, as immuno-deficient populations are more likely to succumb to disease outbreaks. Serosurveys have been conducted to determine the immune status of populations and to provide evidence of past and ongoing HV infections (130, 131). Seroepidemiological studies in three localities in Florida revealed high anti-ChHV seroprevalences (up to 100%) in both FP and non-FP sea turtles (81, 132). Contrastingly, seropositivity to ChHV-5 was dependent on the tumour status in turtles from Hawaii (133). This variation was mainly attributed to differences in the pathogenicity of ChHV-5 subtypes from the two regions (133). In another report, an epizootic of LETD in confined juvenile green sea turtles resulted in 8 to 38% mortality, thus posing significant conservation and management concerns (98, 134). The LETD impact on free ranging sea turtles has not been investigated; however, seroprevalence rates of 13% and 22% were reported in two studies, respectively (134, 135).

Similarly, HV infections are causing increasingly significant concerns in non-marine chelonians (27, 39). Herpesviruses have been implicated as the cause of severe clinical signs and acute death in terrestrial and freshwater turtles (Table 2) (28, 40, 105, 110, 111, 136). Although, HVs have been associated with latent infections in their natural hosts, infections in young, immunosuppressed or non-adapted hosts could result in the development of significant diseases (137). Therefore, monitoring the disease impact on both wild and captive endangered species has become pertinent. In an epidemiological study conducted in Tennessee and Illinois, USA, 128 of 409 free-ranging eastern box turtles (*Terrapene Carolina Carolina*) tested positive for TeHV-1 using TaqMan quantitative PCR, and the detection rate varied widely between seasons (138). Another study reported 48.3% prevalence of HV infections in endangered populations of bog (*Glyptemys muhlenbergii*), wood (*G. insculpta*), and spotted (*Clemmys guttata*) turtles in the northeastern United States (37). Furthermore, tortoise HVs have been associated with high mortality and morbidity (104, 113, 139–141). Different HV species were identified to cause the death of a large number of pancake (*Malacochersus tornieri*), Horsfield (*Testudo horsfieldii*), Hermann's (*Testudo hermanni*), and Egyptian tortoises (*Testudo kleinmanni*) during spontaneous outbreaks in Japan, Italy and Germany, respectively (99, 102, 142). Species dependent susceptibility to HV was reported in a tortoise colony in which *T. graeca* and *T. horsfieldii* appeared to be unaffected by the HV species that caused the death of other tortoises in the same colony (113). A possible explanation could be that the causative HV species is well-adapted in these tortoises and they could be transmitting the virus to naïve or non-adapted tortoises. Of the four tortoise HV species (TeHV1-4), TeHV-3 appears to be the most pathogenic and frequently described, causing lethal disease in different tortoise species (14, 142–145). In a recent assessment of the incidence of chelonian HVs in Europe, more than half (54%) of all the detected chelonian viruses were TeHV-3 (146). Again, seroprevalence rates of 27% and 31% were reported for TeHV-1 and TeHV-3, respectively, in different populations of desert tortoises in California (147, 148). Despite the significance of HV infections, we observed that the disease is still grossly under-studied in some countries (Table 1). Thus, insufficient data and underreporting have made it difficult to assess the geographical patterns of the HV epidemiology in non-marine chelonians and other reptiles.

Herpesviruses have also been described in various species of squamates and crocodilians (25, 47, 48, 52, 106, 116–118, 149). A recent outbreak of a lethal HV infection in a private facility housing 127 snakes resulted in the death of all 71 horned vipers at the premises after a brief illness (150). An earlier study also implicated HV in the death of some boa constrictors within the first year of life (151). Herpesvirus-induced deaths have been reported in different species of lizards with case fatalities nearing 100% (33, 49, 152). As stated earlier, HV infections in crocodiles are associated with CP, SLPE and LNS syndromes (53). Another study strongly linked HV infection to SLPE and CP syndromes in farmed Australian saltwater crocodiles, with the highest prevalence rates of 94 and 54%, respectively (107). Crocodiles are intensively farmed for commercial purposes in Australia; therefore, the occurrence of HVs in crocodiles has both epidemiological and economic implications (53, 153).

Finally, we extracted a total of 130 articles, of which 39% (51 articles) and 32% (41 articles) were studies that investigated HVs in marine turtles and tortoises, respectively. A total of 21 (16%) studies investigated HVs in freshwater turtles. HVs were least studied in lizards (8%; 11 articles), snakes (3%; 4 articles) and crocodiles (2%; 3 articles) (Figure 2). The scant studies of HVs in some reptilian species since the 1970s could be attributed to the unavailability of reagents or sensitive diagnostic assays required to investigate reptilian diseases in remote areas or the lack of interest to investigate HVs in reptiles because of their relatively low socio-economic importance. Therefore, future efforts should be directed towards enhancing collaborations among government agencies, researchers and wildlife workers with a view to creating awareness, increasing access to reagents and sensitive assays, and ultimately conserving endangered reptiles.

## Diagnosis

A timeline of reptilian HV diagnosis showed that traditional assays including histopathology, virus isolation (VI) and electron microscopy (EM) have been the mainstays in the diagnosis of reptilian HVs (Supplementary File 4). Many studies have reported the use of these techniques since the 1970s for the investigation of reptilian HVs. A breakdown of the number of studies that have used these methods to detect reptilian HVs is shown in Figure 3. Molecular diagnosis of reptilian HVs started two decades ago and has been used increasingly since then (Figure 3; Supplementary File 4). The advent of molecular diagnostic techniques has provided insight into the genetic characteristics and the phylogenetic relationship of most reptilian HVs. This section highlights some important characteristics of the various techniques used in the diagnosis of reptilian HVs.

Diagnosis of reptilian HVs is tentatively made on the basis of patient history, clinical signs, and gross and histological lesions (16). However, this is not always the case, as host-adapted HVs can cause subclinical, mild or latent infections in their natural hosts, and the demonstration of intranuclear inclusions is not pathognomonic of reptilian HV infections (154). Intranuclear inclusions are frequently associated with other reptilian viruses including adenoviruses and papillomaviruses (155–157). Earlier researchers used EM to confirm the presence of reptilian HV infections by demonstrating the ultrastructure of the viral particles in fixed, cut and stained sections of tissue samples (51, 54, 98, 106, 116, 136, 140, 158). More recently, EM has been used to confirm a necrotic hepatitis associated with HV infection in a tortoise with no clinical signs or lesions in the respiratory tract, oral cavity or other organs (112). The need for high technical capacities and the high cost of electron microscopes limit the use of EM for epidemiological and diagnostic purposes especially in resource-limited areas. Despite these limitations, EM remains a powerful detection tool in most high-class virology laboratories.

Reptilian HVs have been isolated in cell culture and identified on the basis of their cytopathic effects (50, 98, 107, 159). For instance, tortoise HVs were isolated from pharyngeal swabs, trachea, kidney, oesophagus, tongue, stomach, and intestine, and caused cytolysis and rounding of cells in terrapene heart cells (TH-1) (160). In another study, detachment and foci of enlarged, rounded, refractile cells were produced following inoculation of tissue and swab supernatants in turtle heart cells (142). ChHV-5, which historically has been resistant to replication in conventional cultures, produced *de novo* ballooning degeneration and eosinophilic intranuclear inclusion in plugs and organotypic skin cultures (89). This observation implies that ChHV-5 remains latent in conventional cultures and requires replication of the turtle skin to grow *in vitro* (89). Aside from the fact that CPE are not obtained for non-cytopathic viruses, cell culture is susceptible to both chemical and biological contaminations, which in turn affect its sensitivity and specificity. Also, diagnostic turnaround could be delayed for slow-growing viruses. Therefore, it should not be solely relied upon for the epidemiological investigations of HVs.

Following primary infections in reptiles, a strong non-specific (innate) immune response that includes lysozymes, leukocytes, natural antibodies (NAbs), antimicrobial peptides, and the complement pathway, is quickly stimulated (126, 161). No specific information is currently documented about adaptive cell mediated immunity to HV infections. Unlike mammals, in reptiles a less robust and slower humoral response (IgA, IgD, IgM, and IgY) is stimulated after the innate immune system is activated (126, 154). In tortoises, neutralising antibodies to HV infection were detectable in serum at least 4 weeks post-exposure (162). These serum neutralising antibodies did not appear to confer immunity to reinfection or recrudescence (78). Later seroconversion was observed (four months to one year) in green turtles (*Chelonia mydas*) that were experimentally infected with ChHV (81, 132). Generally, the detection of anti-herpesvirus antibodies in a single sample could indicate previous or latent infection, while rising antibody titre in paired samples collected at least 6 weeks apart indicates active infection (154, 163). Humoral antibodies are detected by serological assays such as serum neutralisation (SN) tests, ELISA, and immunoperoxidase (IP) assays (132, 164–166). The SN test is considered the reference test for anti-herpesvirus antibody detection but has limitations such as a delayed turnaround, inherent assay arduity and the requirement for standard isolates (162). ELISAs with high sensitivity and specificity have been developed and deployed in various seroepidemiological studies (44, 81, 132, 147, 162, 166). However, a high degree of cross-reactivity that potentially affects assay specificity has been demonstrated among different tortoise HV isolates used as antigens in the ELISA (147, 162). Cross-reactivity could also occur in other reptilian HVs that share similar antigenic epitopes, giving false positive results and, thus, leading to unnecessary post-exposure interventions. Overall, serological diagnostic techniques are not useful for the early diagnosis of reptilian HVs because of the delay in antibody response and the need for paired serum sample collection weeks apart with accurate timing. However, it can play an important role in retrospective studies and in the diagnosis of latent or asymptomatic patients.

Recent epidemiological studies have largely relied on molecular methods to identify potential genetic and environmental risk factors associated with reptilian HVs (24, 80, 138, 167–169). Species-specific PCR-based assays targeting specific gene segments of reptilian HVs have been developed and validated (78, 170). Lindeman et al. developed two quantitative PCR assays and recorded a detection limit as low as 1 viral copy per reaction using primers that targeted the EBHV-1 specific segment of DNA polymerase gene (U_L_30) (39). In another study, two TaqMan PCR assays developed to target the U_L_30 gene of TerHV-1 detected 10 viral copies per reaction (171). Conventional and heminested PCR assays using tortoise HV-specific primers have been developed with assay sensitivity of 10^3^ and 10^1^ DNA copies, respectively (172). Alternatively, consensus PCR techniques developed by VanDevanter et al. have been employed for the molecular screening and novel detection of reptilian HV species (39, 43, 100, 173–177). Although the molecular assays for the diagnosis of reptilian HVs have demonstrated excellent performance, their use still presents a major challenge in remote areas due to high cost, complexity of instrumentation, aseptic technique requirement and the need for electricity to operate PCR machines.

In order to accurately estimate the magnitude and scope of a disease outbreak or occurrence, case definition (that is, standard criteria for categorising diseases) would need to be established. One of the ways to achieve this is to make available rapid, sensitive and affordable assays for confirming the presence of diseases. Rapid diagnostic immunoassays that use lateral flow or chromatographic strategies should be developed for the rapid diagnosis of reptilian HV infections in the field or point of care (POC) settings. This approach could overcome some of the above-mentioned diagnostic challenges, especially in low resource areas. However, the use of lateral flow immunoassays for viral detection in other species have been marred by low and varying sensitivities (178–181). Sensitive molecular-based rapid assays are relatively expensive and yet to be employed for the diagnosis of reptilian HVs (182–185). We would propose an ultrasensitive format that combines PCR and immunoassay but then it can be argued that such a laboratory-based system is less rapid and has limited use in low-class laboratories (186, 187). Rapid detection techniques such as Microfluidic chip immunoassay and Smartphone-based rapid telemonitoring system (SBRTS) are fast becoming powerful tools in the diagnosis of viral infections (188–196). Of particular interest, is the SBRTS that combines biosensor and smartphone functionalities to produce a rapid, sensitive and cheap detection system (197). SBRTS has an average turnaround of 30 min, overcomes inherent problems associated with sample handling and preparation, and can remotely monitor and report data on disease occurrence, thus making it suitable for use in resource-limited countries (193, 197). This assay if employed could tick all the boxes for the epidemiological investigation and reporting of reptilian HVs.

Herpesvirus diagnostic and epidemiological data should be interpreted with prudence because of the possible influence of coinfection variables that could cause the reactivation of seemingly latent HV infections. For instance, some studies have reported the detection of co-pathogens in reptiles showing clinical signs, some of which are typical of HV infections (27, 38, 146, 168, 173, 198, 199). These observations imply that the detection of HVs may not be the actual cause of the current disease, but because the immune system is compromised by other pathogens, the HVs recrudesce and become easier to detect. Both latency (decreases apparent prevalence and significance) and coinfections (increase apparent prevalence and may also falsely assign the clinical signs to the HV) will have an influence on the disease picture. Therefore, we recommend that biosecurity and conservation measures should include a multiplex pathogen detection model whenever possible in order to fully assess the health of reptilian populations.

## Treatment, Prevention, and Control

Surgical excision, carbon dioxide (CO_2_) laser surgery and cryosurgery are some of the commonly used therapeutic strategies for the management of HV-associated tumours (25, 110, 111, 120, 200–202). High rates of recurrence and the risk of secondary bacterial infections have greatly reduced the efficacy of surgical excision (200, 203). CO_2_ laser surgery, which combines laser excision and ablation of tumours, has shown improved intraoperative and postoperative outcomes and is therefore the treatment of choice (120, 201, 204). Non-surgical approaches including electrochemotherapy (ECT) and photodynamic therapy (PDT) with no known recurrence have recently been employed as alternatives in the treatment of FP (205, 206).

Several authors have recommended the use of acyclovir complemented by fluid and antibiotic therapies for the effective treatment of tortoise HV infection (143, 207–209). Marschang et al. showed that acyclovir and ganciclovir effectively inhibited HV replication *in vitro* at a single dose or repeated daily dose of 25 or 50 μg/mL (142). Similarly, the *in vitro* activities of acyclovir and ganciclovir were recently tested and shown to be effective against TeHV-3; however, the safety of these drugs is yet to be demonstrated in tortoises (210). Based on the toxicity (on liver and kidney cells) and other biochemical data, this same study showed that eprociclovir is not suitable for use as anti-TeHV-3 in Hermann's tortoises and further *in vivo* assessment of other potential antiviral drugs was recommended (210).

Recently, an autogenous vaccine therapy was proposed and used for the treatment of HV-associated papillomatosis in Williams' mud turtle (*Pelusios williamsi*) (111). The autogenous vaccine, which was aseptically prepared from excised fresh tissue induced substantial areas of necrosis of the papillomatous lesions, thus indicating the efficacy of the vaccine (111). Autogenous vaccines potentially contain relevant neoantigens that comparatively improve their efficacy (211). However, their use could be limited by lack of sufficient tumours (in patients) needed to produce adequate vaccine doses. Also, no standard protocol exists for autogenous vaccine production and delivery, and patients' tumours may progress beyond the intervention stage before the vaccine becomes ready for delivery. Allogeneic vaccines on the other hand, can overcome some of the aforementioned challenges; however, they may lack the advantageous self-neoantigens (211). In the past, an inactivated vaccine was evaluated against tortoise HV without success as no significant rise in antibody was detected in vaccinated tortoises after 369 days post vaccination (160). DNA or mRNA based vaccines have the capacity to induce both humoral and cellular immune responses and have shown promising outcomes against some animal and human diseases (212–215). Although vaccine research and development could be costly, laborious and time-consuming, the nucleic acid vaccines hold the potential to significantly reduce HV-associated losses in captive collections and wild reptiles of conservation concerns.

Prevention is of utmost importance in the management of reptilian HV infections, since death may still occur following therapeutic interventions and recovered animals remain latent carriers (143, 163). Unfortunately, there are no established preventive or control measures for HV infections in wild populations of reptiles (200), which consequently presents a major conservation challenge. Environmental factors including degraded water quality caused by pollutants, increased water temperature, natural biotoxins, and high dietary arginine concentrations due to microalgae bloom have arguably been linked as cofactors in the development of FP in sea turtles (73, 119, 216–221). Therefore, adopting conservation actions needed to regulate water and species management, as well as regulating human activities leading to climate change, would be sensible.

In captive reptiles, quarantine procedures and adequate testing of new acquisitions are strongly recommended (153, 163, 167). All previously infected or HV seropositive animals should be treated as latent carriers and potential shedders to naïve populations, as factors including stress, bad husbandry, illness or immunosuppression could reactivate the virus (14, 163, 167, 200). Generally, strict hygiene practises and adequate biosecurity should be followed in all facilities housing reptiles (162, 172, 222–224).

## Author Contributions

GO and EA conceived and designed this review. GO wrote the manuscript, analysed the data, and prepared figures and tables. EA, PH, and DW contributed to the concept and reviewed drafts of the manuscript. All authors contributed to the article and approved the submitted version.

## Conflict of Interest

The authors declare that the research was conducted in the absence of any commercial or financial relationships that could be construed as a potential conflict of interest.

## Supplementary Material

Supplementary File 1 Literature review search terms and strategies.

Supplementary File 2 Data extraction for literature review.

Supplementary File 3 Co-occurrence map of all keywords from 1975 to 2021. We found a total of 93 keywords with a minimum of 5 occurrences. Node size represents the number of occurrences of each keyword. The lines denote the total link strength between keywords, and it is proportional with the width. The line colours denote five different keyword clusters. The clusters gave an indication of the main areas research interest of reptilian HVs.

Supplementary File 4 A timeline of reptilian HV diagnosis. A review of articles conducted from 1972 to 2020 showed that VI, EM, histopathology, and serologic assays have been used for the detection of reptilian HVs since 1972. Nucleic acid detection assay (PCR) was first reported in 1998 for detection of ChHV-5 in fibropapillomas. Events were chosen based on information from the extracted articles (Supplementary File 2).
