# Supplementary material for: Herpesviruses in Reptiles
Source: Front Vet Sci. 2021 May 5;8:642894. doi: 10.3389/fvets.2021.642894 (PMC8131531; doi:10.3389/fvets.2021.642894)
Supplement: Supplementary File 1 — Literature review search terms and strategies. [file Data_Sheet_1.docx]

**Literature Review Search Terms and Strategies**

1. **Terms searched on Medline and Scopus**
2. Fibropapillomatosis OR grey-patch disease OR herpesviridae infections OR herpesvirus infection OR loggerhead genital-respiratory herpesvirus OR herpesvirus disease AND Reptiles
3. Fibropapillomatosis OR grey-patch disease OR herpesviridae infections OR herpesvirus infection OR loggerhead genital-respiratory herpesvirus OR herpesvirus disease AND Turtles
4. Fibropapillomatosis OR grey-patch disease OR herpesviridae infections OR herpesvirus infection OR loggerhead genital-respiratory herpesvirus OR herpesvirus disease AND Tortoise
5. Fibropapillomatosis OR grey-patch disease OR herpesviridae infections OR herpesvirus infection OR loggerhead genital-respiratory herpesvirus OR herpesvirus disease AND Snakes
6. Fibropapillomatosis OR grey-patch disease OR herpesviridae infections OR herpesvirus infection OR loggerhead genital-respiratory herpesvirus OR herpesvirus disease AND Alligators and Crocodiles
7. Fibropapillomatosis OR grey-patch disease OR herpesviridae infections OR herpesvirus infection OR loggerhead genital-respiratory herpesvirus OR herpesvirus disease AND Lizards
8. **Terms searched on PubMed and Scopus**
9. Herpesvirus reptiles
10. Herpesvirus turtles
11. Herpesvirus tortoise
12. Herpesvirus Snakes
13. Herpesvirus alligators and crocodiles
14. Herpesvirus lizards
15. Fibropapillomatosis reptiles
16. Grey patch disease reptiles
17. Loggerhead genital-respiratory herpesvirus reptiles
