# Supplementary figures and images for: Herpesviruses in Reptiles
Source: Front Vet Sci. 2021 May 5;8:642894. doi: 10.3389/fvets.2021.642894 (PMC8131531; doi:10.3389/fvets.2021.642894)

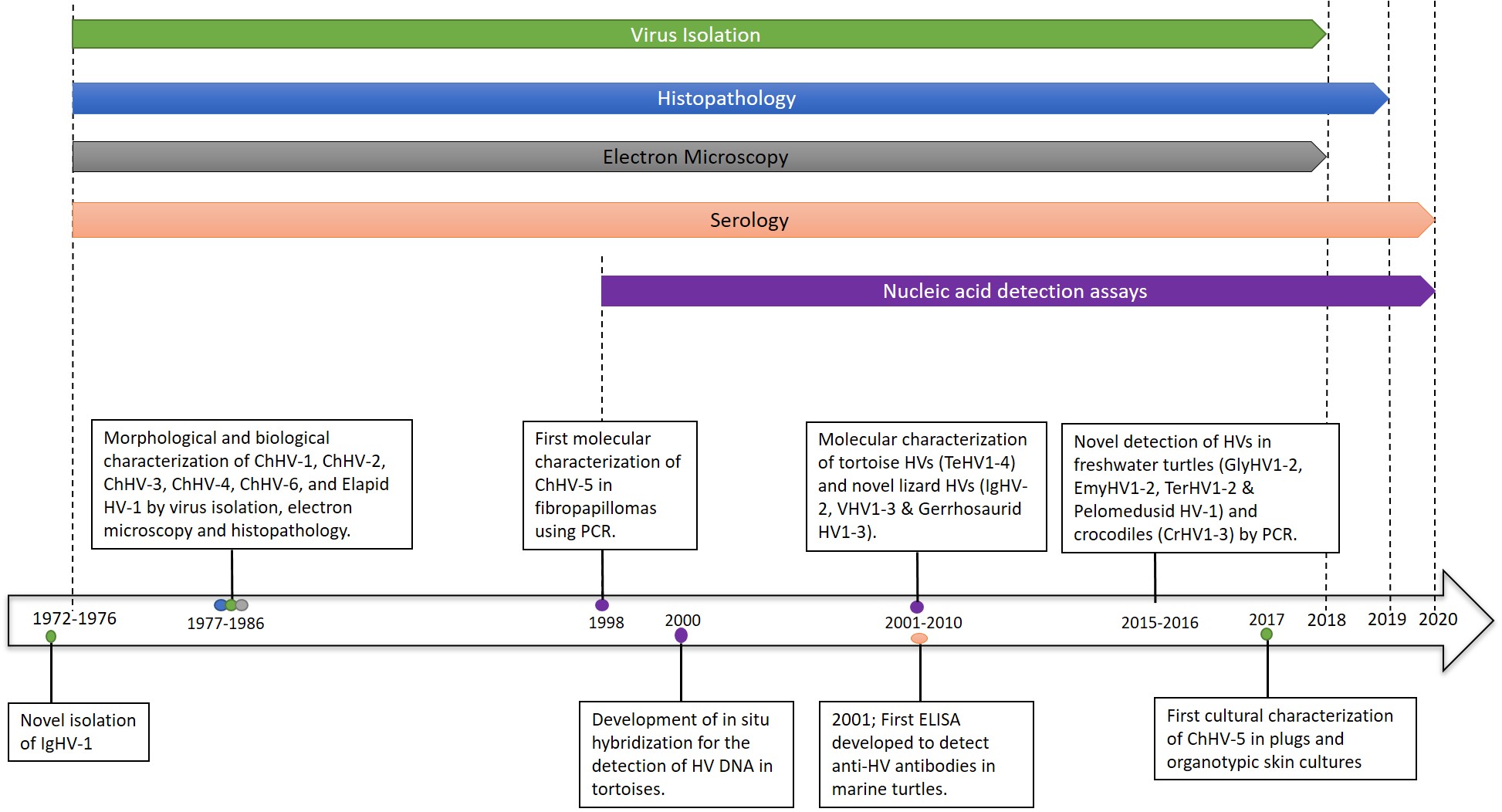

Supplement: Supplementary File 3 — Co-occurrence map of all keywords from 1975 to 2021. We found a total of 93 keywords with a minimum of 5 occurrences. Node size represents the number of occurrences of each keyword. The lines denote the total link strength between keywords, and it is proportional with the width. The line colours denote five different keyword clusters. The clusters gave an indication of the main areas research interest of reptilian HVs. [file Image_2.JPEG]

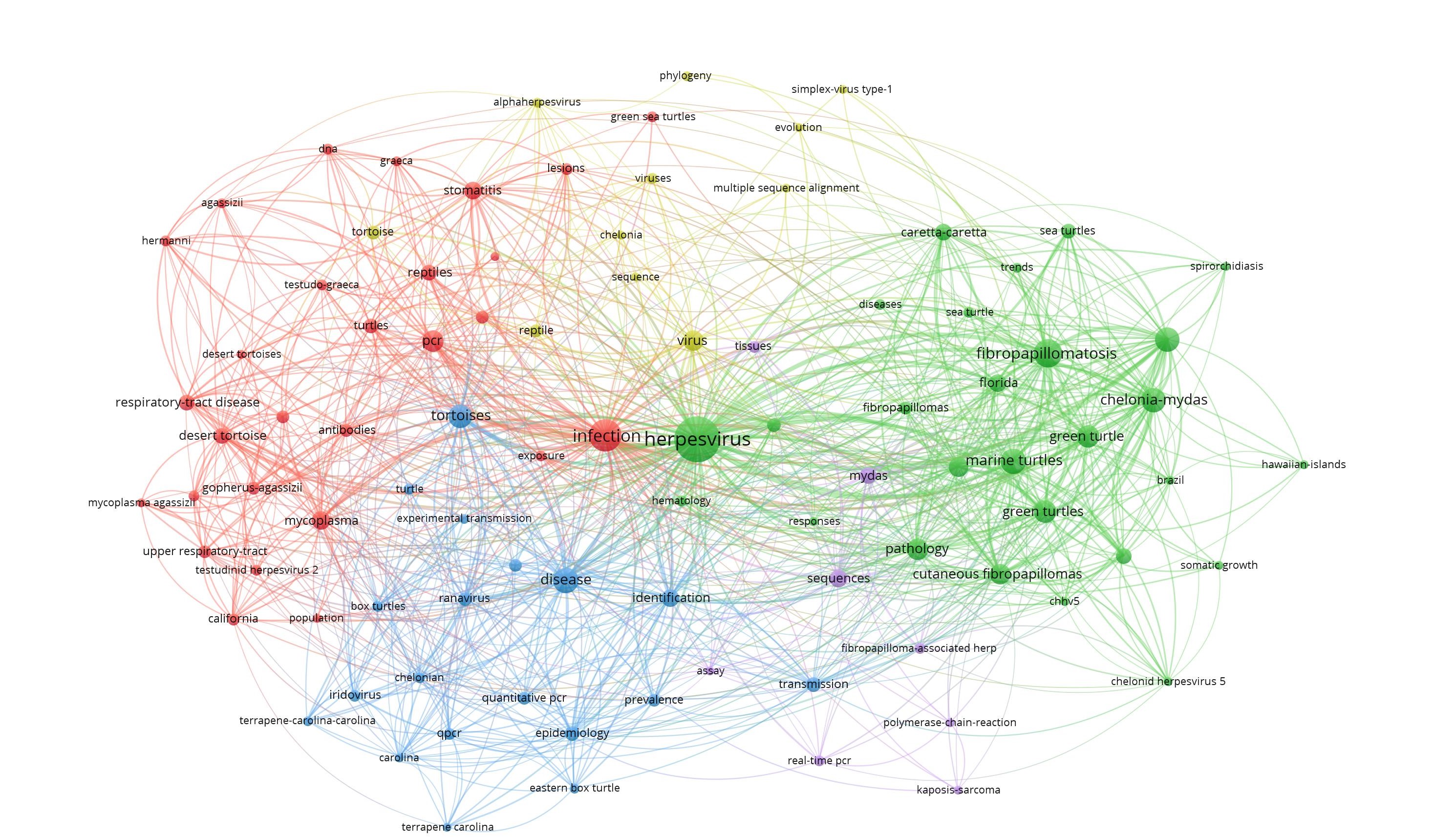

Supplement: Supplementary file 4 [file Image_1.JPEG]
